# Pseudoaneurysm: A complication of laparoscopic inguinal hernia repair

**DOI:** 10.1016/j.ijscr.2018.11.045

**Published:** 2018-11-27

**Authors:** Brijendra Singh, Vaibhav Gupta, Shahana Gupta

**Affiliations:** a The Department of Surgical Gastroenterology, Gastro Liver Hospital, 7/190 D- 1,2,3, Swaroop Nagar, Kanpur, 208002, Uttar Pradesh, India; b Department of Surgical Gastroenterology, Medical College, Kolkata, India

**Keywords:** Pseudo aneurysm, Laparoscopic inguinal hernia repair, Complications of laparoscopic inguinal hernia repair, Case report

## Abstract

**Introduction:**

Laparoscopic inguinal hernia repair has gained importance during last few decades, because of its several advantages. However, it has its own set of complications, which can be avoided by following the basic anatomical principles of laparoscopic inguinal hernia repair.

**Presentation of case:**

An 85-year-old male patient, developed a painless intraabdominal lump following laparoscopic inguinal hernia repair. Clinical and radiological evaluation was suggestive of pseudo aneurysm. Intra operative findings revealed a pseudo aneurysm arising from the right deep circumflex iliac artery. Excision of the pseudo aneurysm with incorporated mesh along with orchidectomy followed by fascial repair was performed.

**Discussion:**

Pseudo aneurysm refers to a defect in an arterial wall, which allows communication of arterial blood with the adjacent extra-luminal space. Blood extravasates out of the artery, which is contained by surrounding soft tissue and compressed thrombus that forms a cavity or sac. With the increasing utilization of percutaneous arterial interventions worldwide, iatrogenic arterial injury has become the predominant cause of pseudo aneurysm formation. Here we report a case on pseudo aneurysm arising from deep circumflex iliac artery following laparoscopic inguinal hernia repair. This complication can be avoided by following the basic principles of surgical anatomy of the pelvis and groin as viewed through the laparoscope.

**Conclusion:**

Pseudo aneurysm complicating laparoscopic inguinal hernia repair is a rare occurrence. Injury to seemingly small artery can lead to evolution of pseudoaneurysm. Adherence to surgical principle will avoid such a complication.

## Highlights

• Laparoscopic Repair of Inguinal Hernia has gained importance during last few decades because of it’s several advantages, but it is still difficult to comment upon its outcomes.
• One of the least reported complications of laparoscopic repair of inguinal hernia is the pseudo aneurysm formation due to a vascular injury, which can be avoided by following the basic surgical principles and knowledge of the anatomical aspect of the groin region as viewed laparoscopically.
• Pseudo aneurysm complicating laparoscopic inguinal hernia repair is a rare occurence. Injury to seemingly small artery can also lead to evolution of pseudo aneurysm.

## 1 Introduction

Laparoscopic inguinal hernia repair has become one of the most accepted forms of minimal access surgery. The advantages, include less post-operative discomfort and pain, reduced recovery time, easier repair of a recurrent hernia, the ability to treat bilateral hernias concurrently, the performance of a simultaneous diagnostic laparoscopy, ligation of the hernia sac at the highest possible site and improved cosmesis. But as any other surgical method it also has its own set of complications i.e. recurrence, infection, neuro-vascular injury, visceral injury, injury to vas and other testicular complications. Here we present a case of pseudo aneurysm formation which could have been avoided by following the basic laparoscopic inguinal hernia anatomy and the principles of the repair. The work has been reported in line with the SCARE criteria [1].

## 2 Presentation of case

**Fig. 1:**
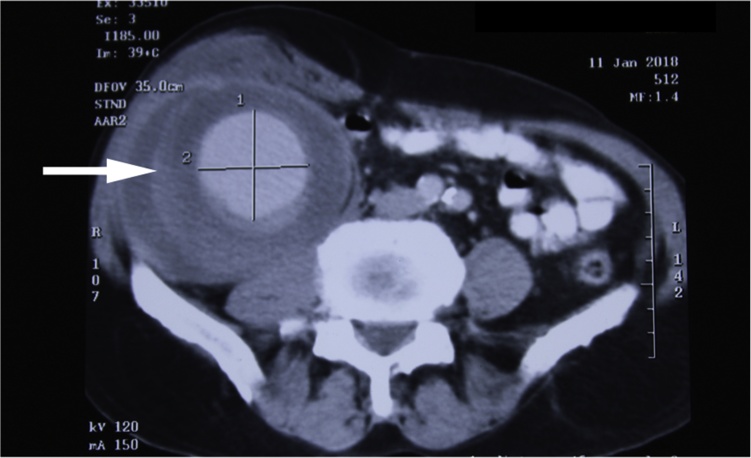
CECT image – Arrow showing hypodense lesion in right iliac fossa, enhancing with intravenous contrast.

**Fig. 2:**
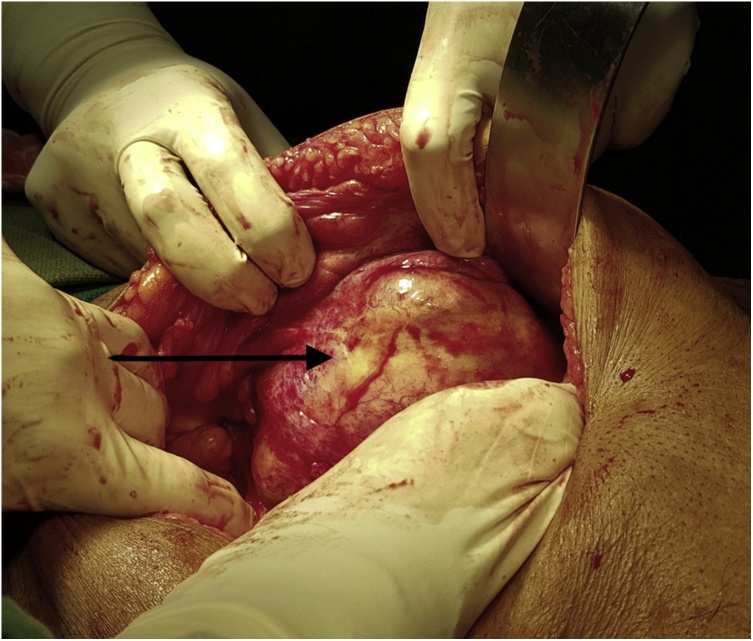
Intra operative image – Arrow showing the cystic swelling.

**Fig. 3:**
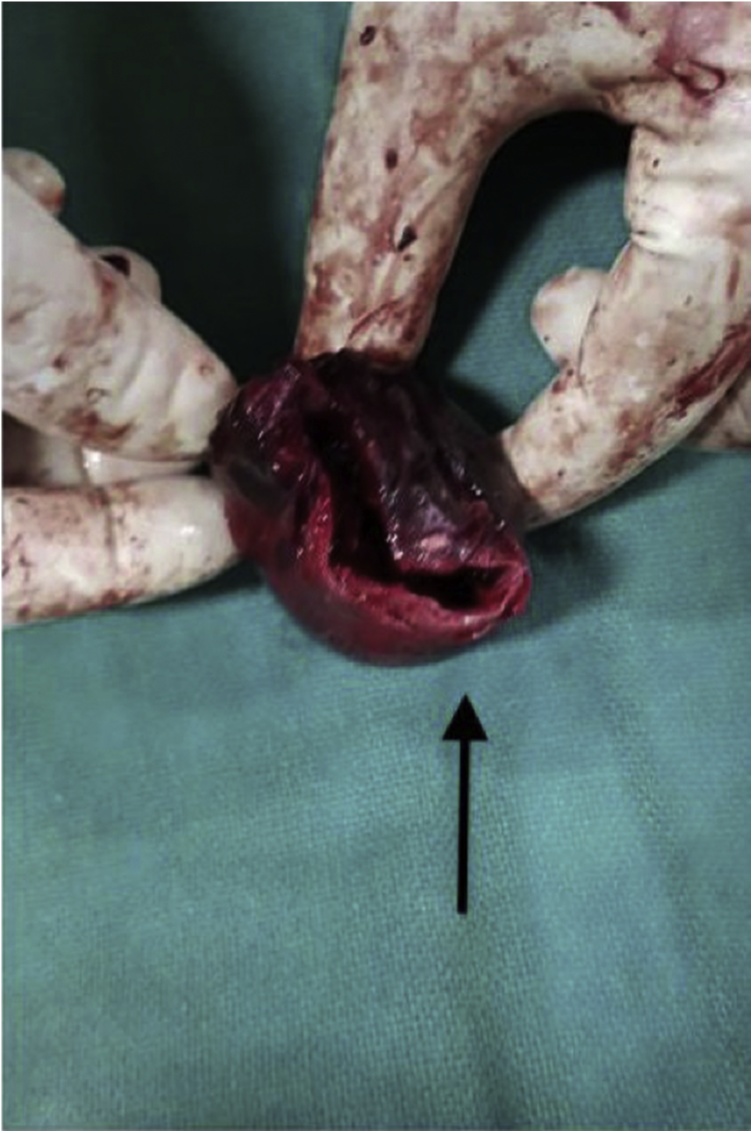
Per operative image – Arrow showing the excised swelliing with incorporated previous mesh.

An 85-year-old male patient presented with complaints of right sided intra-abdominal lump since last six months. The lump was painless but progressive and not associated with any other symptoms. The patient underwent a right sided laparoscopic inguinal hernia repair elsewhere (*trans* abdominal pre-peritoneal repair), about one year back. On examination, the patient was hemodynamically stable, per abdomen findings were suggestive of a non-tender, large, intra-abdominal cystic, pulsatile swelling in the right iliac fossa and the right lumbar region, measuring approximately 5 × 7 cm. CECT abdomen showed, a hypodense lesion in the right iliac fossa, enhancing with intravenous contrast (Fig. 1). Laparotomy was performed. Intra operative findings revealed a pseudo aneurysm arising from the right deep circumflex iliac artery (Fig. 2). Prolene mesh of previous repair was also incorporated in the wall of the lesion. Hence, we performed an excision of the pseudo aneurysm with incorporated mesh along with right orchidectomy followed by fascial repair (Fig. 3). Post operatively, patient made an uneventful recovery. Biopsy was compatible with diagnosis of pseudo aneurysm.

## 3 Discussion

A pseudo aneurysm refers to a defect in an arterial wall, which allows communication of arterial blood with the adjacent extra-luminal space. Blood extravasates out of the artery but is contained by surrounding soft tissue and compressed thrombus [2]. Pseudo aneurysms are typically the result of traumatic arterial injury.

With the increasing utilization of percutaneous arterial interventions worldwide, iatrogenic arterial injury has become the predominant cause of pseudo aneurysm formation. Here we report a case on pseudo aneurysm arising from deep circumflex iliac artery (DCIA) following laparoscopic inguinal hernia repair. The reason for pseudo aneurysm formation in this case could have been the use of tackers to fix the mesh. This complication can be avoided by following the basic principles of surgical anatomy of the pelvis and groin as viewed through the laparoscope. The use of electrosurgical energy, dissection, or the application of staples or tackers within the danger zones i.e. triangle of doom and triangle of pain should always be avoided.

Iatrogenic pseudo aneurysm of the DCIA is a rare entity. A review of literature revealed 11 reports of pseudo aneurysms, which include anterior iliac bone graft harvesting [3], surgical drain placement [4], guide-wire injury during femoral artery catheterization [5], paracentesis [6]. However, we found minimal literature on pseudo aneurysm arising from DCIA following laparoscopic inguinal hernia repair.

Pseudo aneurysms develop after any event that causes partial disruption of the vessel wall. The anatomical location of the DCIA, especially its ascending branch, within the anterior abdominal wall, which makes it vulnerable to injury during abdominal wall procedures. DCIA originates from the external iliac or femoral artery in the region of inguinal canal, just deep and superior to the inguinal ligament. Then it courses laterally and upward toward the medial aspect of the anterior superior iliac spine. As it approaches the anterior superior iliac spine, it gives off a large ascending branch, which pierces the transversus abdominis muscle and lies between it and the internal oblique muscle. The artery then leaves the inguinal floor, pierces the transversalis fascia and enters a fibro-osseous tunnel formed by the line of attachment of the transversalis fascia and the iliacus fascia.

The highest incidence of iatrogenic pseudo aneurysm formation is observed in the common femoral artery as a result of inadequate seal of the arterial puncture site following catheterization procedures [7]. The incidence of pseudo aneurysm formation is approximately 1% with diagnostic studies but increases to 3.2% when an interventional procedure is performed. The incidence increases with multiple catheterization procedures on the same vessel [8,9]. Due to its rarity, the diagnosis may be missed and potential complications, including, persistent painful swelling, development of an abscess or rupture of a pseudo aneurysm may ensue in an untreated patient.

Pseudo aneurysms can enlarge and produce local compressive symptoms, erode adjacent structures or rarely be a source of distal emboli. They can initially be clinically occult but with time become symptomatic.

This is a rare but dangerous complication noted following a laparoscopic inguinal hernia repair. One should be very well acquainted with the anatomy of the groin region as viewed through the laparoscope, and always follow the basic principles of repair.

## 4 Conclusion

Laparoscopic repair of inguinal hernia is gaining the importance because of significant advantages. One of the least reported complications is the pseudo aneurysm formation due to a vascular injury. This rare complication can be avoided by following basic surgical principles and knowledge of the anatomical aspect of the groin region as viewed laparoscopically.

## Conflicts of interest

None.

## Funding

None.

## Ethical approval

This is a case report in which no research has been done. Name of the patient is not disclosed. So ethical approval was not required.

## Consent

Written informed consent was obtained from the patient for publication of this case report and accompanying images. A copy of the written consent is available for review by the Editor-in-Chief of this journal on request.

## Author contribution

Dr. Brijendra Singh: Discussion, Figures.

Dr. Vaibhav Gupta: Abstract, Introduction, Presentation of case, Conclusion.

Dr. Shahana Gupta: Highlights, References.

Ms Eshani Singh: Contributor (Secretarial help).

## Registration of research studies

This is a case report and not a research work. Hence registration not done.

## Guarantor

Dr. Brijendra Singh.

## Provenance and peer review

Not commissioned, externally peer reviewed.
